# Folgoration as an Example of Pathergy in a Patient Affected by Pyoderma Gangrenosum and Takayasu's Arteritis

**DOI:** 10.1155/2009/393452

**Published:** 2009-11-04

**Authors:** A. G. Richetta, S. D'Epiro, C. Mattozzi, S. Giancristoforo, S. Calvieri

**Affiliations:** Department of Dermatology and Venereology Policlinico Umberto I, University of Rome “La Sapienza“ viale del Policlinico 155, 00160 Rome, Italy

## Abstract

Pyoderma gangrenosum (PG) is a neutrophilic dermatosis of unknown aetiology. Clinical manifestations of PG are characterized by destructive, necrotizing, and noninfective ulceration of the skin. 20–30% of cases are initiated and aggravated by minor trauma or surgery, a phenomenon named pathergy. PG is related to several autoimmune diseases including ulcerative colitis, Crohn's disease, rheumatoid arthritis, and monoclonal gammopathy. The association with Takayasu's arteritis (TA), a chronic inflammatory and stenotic disease of large and medium-sized arteries, is instead less common. We report a case of PG associated with TA that was induced by an accident with folgoration of the skin; in this case the folgoration can be considered as an exemple of Pathergy, that is, a characteristic feature of PG.

## 1. Introduction

Pyoderma gangrenosum (PG) is a neutrophilic dermatosis of unknown aetiology. Clinical manifestations of PG are characterized by destructive, necrotizing, and noninfective ulceration of the skin mostly on the lower extremities. 20–30% of cases are initiated and aggravated by minor trauma or surgery, a phenomenon named pathergy [1]. 

 PG is related to several autoimmune diseases including ulcerative colitis, Crohn's disease, rheumatoid arthritis, and monoclonal gammopathy. The association with PG is also a well-known complication of Takayasu's arteritis (TA), a chronic inflammatory, and stenotic disease of large and medium-sized arteries [2]. 

 Most cases of pyoderma gangrenosum associated with Takayasu's arteritis have been observed in Japan, while reports appeare rarely in the Western literature [3–7].

## 2. Case Report

We describe the case of a 75-year-old Caucasian woman presented to our attention for the presence of many esulcerative lesions on the legs, with unruffled limits and violaceus borders. 

**Figure 1:**
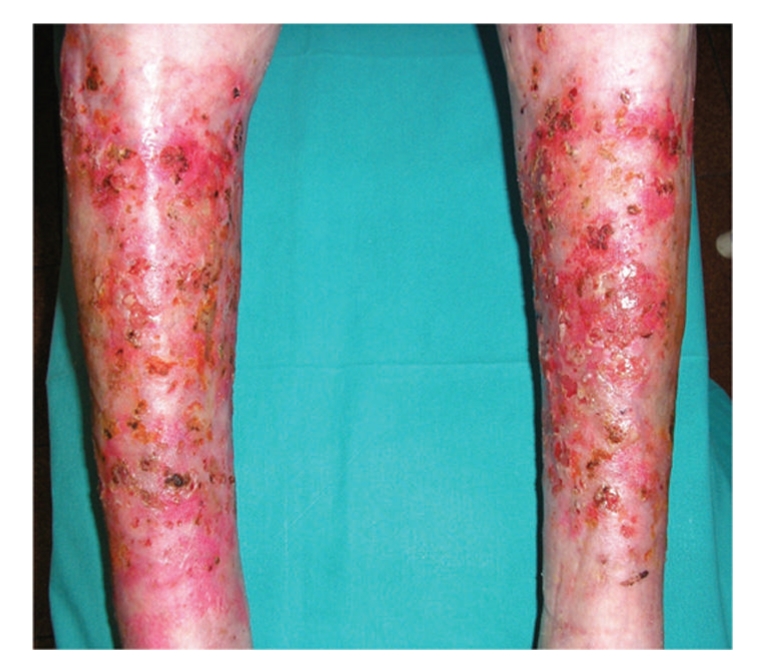
Esulcerative lesions on the legs, with unruffled limits and violaceus borders.

The patient had an accident two months before with folgoration of the skin but without any involvement of interior organs. After the accident she was submitted to a surgical intervention of autologous skin grafting but it was a failure, obtaining only a partial taking root (Figure 1).

The patient referred to be affected by TA, diagnosed 28 years before. 

 At the physical examination she presented an asymmetry of radial arterial pulse, with diminished left arterial pulse intensity. 

 As the patient presented to our attention, we firstly made a bacterial swab of skin ulcers which responce showed an infection with *Staphylococcus* *aureus*, while serological tests revealed a state of inflammation; the patient underwent to antibiotic therapy with Minocyclin 100 mg twice a day for 10 days. 

 Local wound care was based on the use of collagen, silver and nonadherent medications, but we did not obtain any improvement of the skin lesions. 

**Figure 2:**
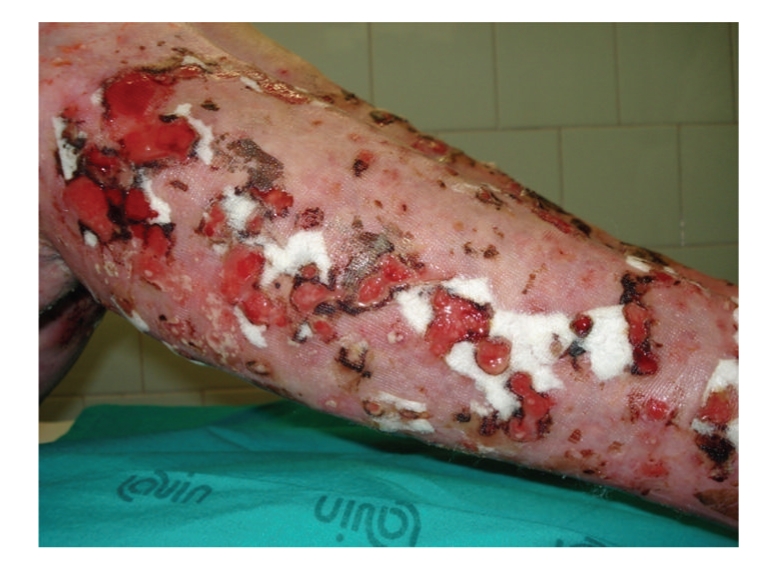
Pustules gradually involving in esulcerative lesions, characterized by a progressive external expansion, unruffled limits and violaceus borders.

 Even more during time new lesions began to appeare, starting as pustules and gradually involving in esulcerative lesions, characterized by a progressive external expansion, unruffled limits and violaceus borders (Figure 2). 

 Then we decided for submitting the patient to a skin biopsy, which histopathologic examination showed a severe inflammation with a dense neutrophilic infiltrate in the dermis. The clinical features and the histopathological findings were consistent with a diagnosis of PG, so that we decided to initiate a treatment with systemic prednisone 40 mg once a day and a topical corticosteroid applied once daily. 

**Figure 3:**
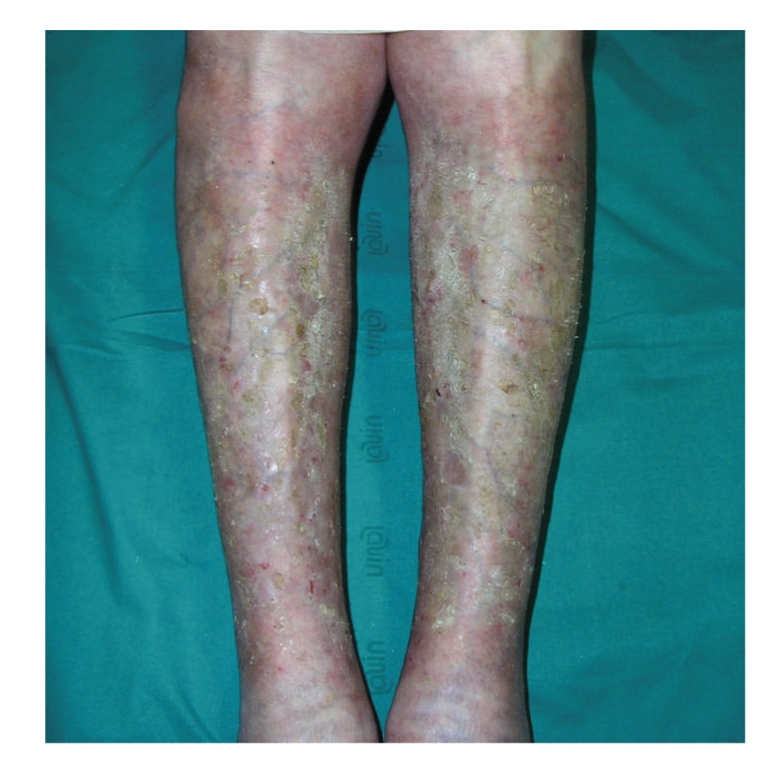
The complete resolution of the skin lesions after one month of therapy.

Within 2 weeks wounds showed marked signs of improvement and systemic prednisone was gradually reduced in a month, obtaining a complete resolution of the skin lesions (Figure 3).

## 3. Discussion

PG is an uncommon ulcerative neutrophilic dermatosis with distinctive clinical characteristics and a frequent association with systemic disease. It is characterized by a progressive and a recurrent skin ulceration. The lesions most commonly occur on the legs, but they may also occur elsewhere. 

The classic form of PG is characterized by a deep ulceration with a violaceous border; the skin lesions are painful, present with undermined edges and a necrotic base, and often go rapidly enlarging. The atypical form has a vesiculo-pustular component [1]. Disorders classically associated with pyoderma gangrenosum include inflammatory bowel disease, rheumatoid arthritis, paraproteinemia and myeloproliferative disorders; the association with TA is less common.

Takayasu's arteritis is a large vessel vasculitis characterized by stenosis or occlusion of the aorta and its branches, it usually affects young women and it is common in Japan, South-east Asia, India, Middle and South American countries. Its aetiology is unknown. Cutaneous manifestations have been estimated to affect between 2.8% and 28% of patients, depending on race [8, 9]. They include erythema nodosum in Europe and North America, pyoderma gangrenosum in Japan, ulcerated nodular lesions, erythema induratum, papulonecrotic eruptions, popular erythematous lesions on the hands and feet, facial eruptions resembling lupus erythematosus, postgranulomatous anetoderma, erythema multiforme, urticaria, cutaneous polyarteritis nodosa, granulomatous vasculitis and Raynaud's phenomenon [3–7]. 

In Europe the mean age of presentation is 41 years and women are more commonly affected than men [10]. 

 PG is a well-known complication of Takayasu's arteritis (TA) in Japan, while this association is very rare in North America and Europe, so that this condition has not been fully described in literature [2]. 

 The features of PG are not specific histopathologically and for this reason diagnosis is often based on clinical feature [11]. Our patient had clinical signs of PG that were consistent with the pustular variant of PG; our diagnosis was also supported by the histological features of the affection.

The wounds showed no signs of healing despite therapy with systemic antibiotics and local wound care, supporting the diagnosis of PG. 

We have reported a case of PG associated with TA, developed after an accident with folgoration of the skin. In this case the folgoration may be considered an example of Pathergy, that is the initiation of new lesions from sites of previous trauma. 

Pathergy is a characteristic feature of PG. It occurs in 20–30% of patients with PG and it may result from minor trauma such as debridement, minor cutaneous surgery or major surgery [1].

To our knowledge this is the first case described in literature of folgoration as an example of induction of PG in a patient affected by TA. 

 We observed a rapid and complete resolution of the skin lesions with oral corticosteroid therapy. 

Corticosteroids are the treatment of choice for pyoderma gangrenosum. In corticosteroid-resistant pyoderma gangrenosum, many other systemic agents such as minocycline and methotrexate have been tried with varying success. Intravenous immune globulin has also been used to treat pyoderma gangrenosum, although no reports of its use in Takayasu's arteritis could be found. Cyclosporin has been reported to be effective in severe cases of pyoderma gangrenosum [10]. 

 Corticosteroids are also the treatment of choice for patients with Takayasu's arteritis; however, some patients with monophasic self-limiting disease require no specific treatment [12].

Then, first-line therapy in disseminated PG is systemic treatment with prednisone (0.5–1 mg/kg/day) or cyclosporine (5 mg/kg/day) alone or combined due to their anti-inflammatory and immunosuppressant properties and rapid efficacy [13]. Several studies suggests antiTNF*α* as a promising alternative to the common immunosuppressive treatment regimens [11, 14, 15]. 

 Concluding, it is very important to meditate that when a wound is not healing despite relevant local wound management and systemic treatment if needed, the clinician should always suspect PG. Early diagnosis and treatment is in fact crucial in the management of this skin affection.

It is also very important to remember the rare possibility of underlying Takayasu's arteritis in all patients with pyoderma gangrenosum. 

 Finally, particular attention must be reserved to trigger factors and pathergy as cause of induction or reactivation of PG.
